# Integrated analysis of single‐cell RNA‐seq and bulk RNA‐seq unravels the molecular feature of M2 macrophages of head and neck squamous cell carcinoma

**DOI:** 10.1111/jcmm.18083

**Published:** 2024-02-23

**Authors:** Siyuan Wu, Xiaozhi Lv, Haigang Wei, Jialin Wu, Shiwei Liu, Xia Li, Jing Song, Chen Zou, Yilong Ai

**Affiliations:** ^1^ Foshan Stomatological Hospital School of Medicine, Foshan University Foshan Guangdong China; ^2^ Department of Oral and Maxillofacial Surgery ZhuJiang Hospital, Southern Medical University Guangzhou China; ^3^ Department of Stomatology Foshan First People's Hospital Foshan Guangdong China

**Keywords:** head and neck squamous cell carcinoma, immunity, macrophage, prognosis, single‐cell analysis

## Abstract

The connection between head and neck squamous cell carcinoma (HNSC) and M2 tumour‐associated macrophages is not yet fully understood. We gathered gene expression profiles and clinical data from HNSC patients in the TCGA database. Using Consensus Clustering, we categorized these patients into M2 macrophage‐related clusters. We developed a M2 macrophage‐related signature (MRS) through statistical analyses. Additionally, we assessed gene expression in HNSC cells using single‐cell sequencing data (GSE139324). We identified three distinct M2 macrophage‐related clusters in HNSC, each with different prognostic outcomes and immune characteristics. Patients with different MRS profiles exhibited variations in immune infiltration, genetic mutations and prognosis. FCGR2A may play a role in creating an immunosuppressive tumour microenvironment and could potentially serve as a therapeutic target for HNSC. Our study demonstrated that M2 macrophage‐related genes significantly impact the development and progression of HNSC. The M2 macrophage‐related model offered a more comprehensive assessment of HNSC patient prognosis, genetic mutations and immune features. FCGR2A was implicated in immunosuppressive microenvironments and may hold promise for the development of novel immunotherapeutic strategies for HNSC.

## 1 INTRODUCTION

Head and neck squamous cell carcinoma (HNSC) encompasses cancers affecting the nasal cavity, sinuses, mouth, tonsils, pharynx and larynx.^1^, ^2^ It ranks among the most prevalent malignancies worldwide.^3^ Despite significant advancements in cancer diagnosis and treatment, the 5‐year survival rate for HNSC remains unsatisfactory because of its heterogeneous and aggressive nature.^4^, ^5^, ^6^ Primary treatment modalities for HNSC patients include surgery and chemoradiotherapy; however, 40%–60% of patients face the risk of recurrence.^7^ Therefore, early identification of biomarkers for prognostic purposes is essential to improve the management of HNSC patients.

Tumour‐associated macrophages (TAMs) represent a subtype of immune‐related stromal cells involved in the formation of tumour microenvironment (TME).^8^, ^9^, ^10^ TAMs can facilitate tumour proliferation, invasion and migration across various tumour types and are associated with an unfavourable prognosis.^11^, ^12^ Typically, TAMs are mainly categorized into M1 and M2 macrophages. M1 macrophages can eliminate tumour cells through direct or indirect cytotoxicity, while M2 macrophages promote tumour angiogenesis and facilitate tumour metastasis by suppressing T‐cell‐mediated anti‐tumour immune responses.^13^, ^14^, ^15^ Dysregulation of the M1/M2 polarization balance leads to tumour‐supporting and tumour‐promoting effects of TAMs.^16^ Consequently, M2 macrophages play a pivotal role in tumour progression, although specific mechanisms involving M2 macrophage‐related genes (MRGs) in HNSC remain unexplored.

In conclusion, we conducted an analysis of the prognostic and immunological features of MRGs in HNSC. We delineated distinct M2 macrophage‐associated clusters with specific prognostic and immunological characteristics. Furthermore, we developed a prognostic model based on MRGs using the Cancer Genome Atlas (TCGA) dataset to predict the prognosis of HNSC. Additionally, we validated the expression levels of modelled genes in various cells types using the single‐cell sequencing dataset (GSE139324).

## 2 METHODS

### 2.1 Data acquisition and processing

We obtained the consolidated transcriptome expression matrix and clinical data for HNSC from the TCGA database. Single cell sequencing data of HNSC patients (GSE139324)^17^ were sourced from the Tumour Immune Single‐cell Hub 2 (TISCH2).^18^ We employed an external validation dataset, GSE41613,^19^, ^20^ to corroborate and validate the prognostic features of our model. We collected immune‐related genes from ImmPort portal (https://www.immport.org/home) and InnateDB (https://www.innatedb.ca/).

### 2.2 Cluster analysis

Initially, we employed univariate Cox regression analysis to screen for prognostic MRGs. ‘Consensus Clustering’ is a statistical technique used to identify robust and stable clusters within a dataset. The resulting consensus matrix provides insights into the inherent structure of the data, allowing researchers to identify groups of data points that exhibit similar characteristics. A ‘cluster’ in this context refers to a group of data points that share similar attributes or characteristics. We then applied the Consensus Clustering algorithm to establish M2 macrophage‐related clusters. The differences in prognostic outcomes among these clusters were assessed using Kaplan–Meier (KM) curves. To annotate functional genes and analyse their function, along with advanced genome function information, we conducted Gene Ontology (GO), Kyoto Encyclopedia of Genes and Genomes (KEGG) and Gene Set Variation Analysis (GSVA).

### 2.3 Immune infiltration analysis

To quantitatively assess the relative abundance of each immune cell within the TME, we employed various algorithms. The ‘estimate’ package was utilized to calculate TME scores. Additionally, the ‘ssGSEA’ package was employed to determine immune function scores, which provide enrichment scores for various immune functions. The immune activity scores for HNSC samples were obtained from the tracking Tumour Immunophenotype (TIP, http://biocc.hrbmu.edu.cn/TIP/).

### 2.4 Prognostic model analysis

We developed the M2 macrophage‐related signature (MRS) through a combination of the least absolute shrinkage and selection operator (LASSO) Cox regression and multivariate Cox regression analyses. To assess the predictive accuracy of the MRS, we employed KM curves, receiver operating characteristic (ROC) curve, and performed univariate and multifactorial independent prognostic analysis.

### 2.5 Mutation landscape and drug sensitivity analysis

Mutation data were retrieved from the TCGA database, and the ‘Maftools’ package was utilized to visualize the respective mutational profiles of two risk levels via a waterfall plot. For targeted therapy drug analysis, we employed the ‘pRRophetic’ package to evaluate the half maximum inhibitory concentration (IC50) of nine common HNSC chemotherapy drugs.

### 2.6 Statistical analysis

All analyses were performed by using R 4.1.0. All statistical tests were two‐sided, and *p*‐value <0.05 was considered statistically significant unless otherwise noted. KM curve and Cox regression analysis were utilized to analyse the correlation between clinicopathological features and OS in HNSC patients. The univariate regression model was applied to analyse the effects of individual variables on survival. The LASSO cox regression model was employed to confirm independent impact factors associated with survival. Table S1 documented all abbreviations along with their corresponding full names.

## 3 RESULTS

### 3.1 Construction of the M2 macrophage‐related clusters and biological analysis

**FIGURE 1:**
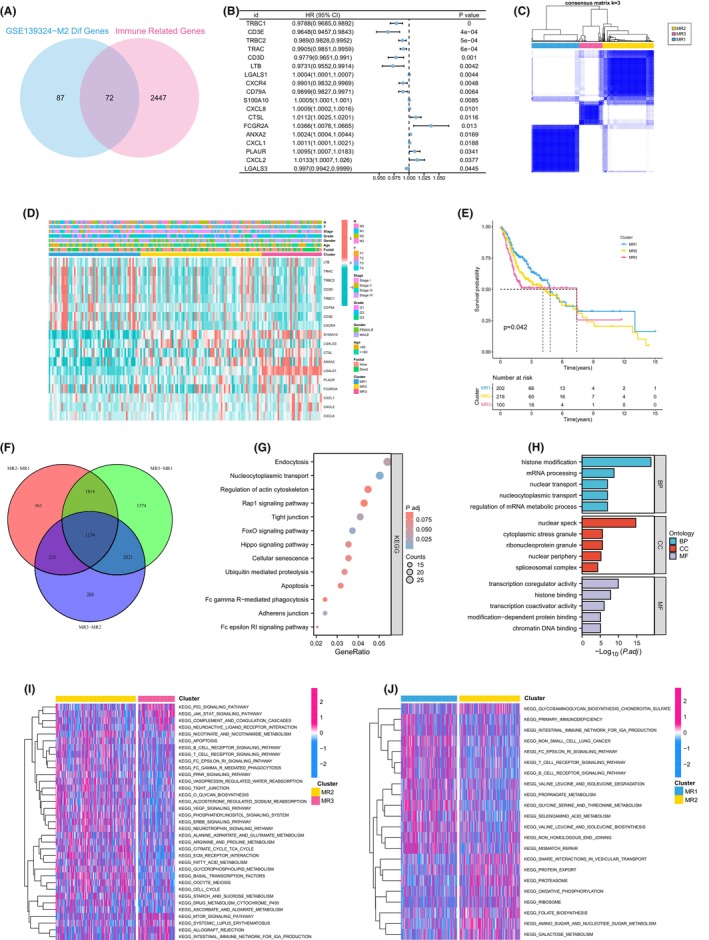
Construction and biological analysis of M2 macrophage‐related clusters. (A) Screening of genes associated with M2 macrophages. (B) Univariate Cox analysis for M2 macrophage‐related genes (MRGs). (C) Distribution of samples among M2 macrophage‐related clusters. (D) Distribution of M2 MRGs and clinicopathologic variables across M2 macrophage‐related clusters. (E) Kaplan–Meier (KM) curve analysis for M2 macrophage‐related clusters. (F) Identification of overlapping differentially expressed genes among the M2 macrophage‐related clusters. (G, H) Results of Kyoto Encyclopedia of Genes and Genomes (KEGG) and Gene Ontology (GO) enrichment analyses. (I, J) Results of Gene Set Variation Analysis (GSVA) enrichment analysis for M2 macrophage‐related clusters.

In the GSE139324 dataset, we identified M2 macrophage differential genes based on an adjusted *p*‐value <0.001 and |log2fc| > 1, resulting in the identification of 72 MRGs that overlapped with immune‐related genes and macrophage differential genes (Figure 1A). Further filtering through univariate Cox analysis (*p* < 0.05) yielded 18 prognostic MRGs (Figure 1B). Based on the expression of these 18 prognostic MRGs, we applied the Consensus Clustering algorithm to classify HNSC patients into three distinct clusters (Figure 1C). The heatmap illustrated the distribution of MRGs and clinical pathological variables across these different M2 macrophage‐related clusters (Figure 1D). KM survival analysis revealed that MR3 had the worst prognosis, while MR1 exhibited the best prognosis (Figure 1E). Additionally, we identified 1174 differentially expressed genes (DEGs) using an adjusted *p*‐value <0.05 and |log2fc| > 1 (Figure 1F). KEGG analysis uncovered DEGs involved in various cellular functions, including nucleocytoplasmic transport, tight junctions, cellular senescence and apoptosis (Figure 1G). GO enrichment analysis demonstrated significant enrichment of DEGs in RNA and histone modification (Figure 1H). Furthermore, GSVA analysis indicated significant enrichment of immunoactivation pathways in MR1, matrix‐related pathways in MR2 and multiple cancer‐promoting pathways in MR3 (Figure 1I, J).

### 3.2 Identification of immune infiltration characteristics of M2 macrophage‐related clusters

**FIGURE 2:**
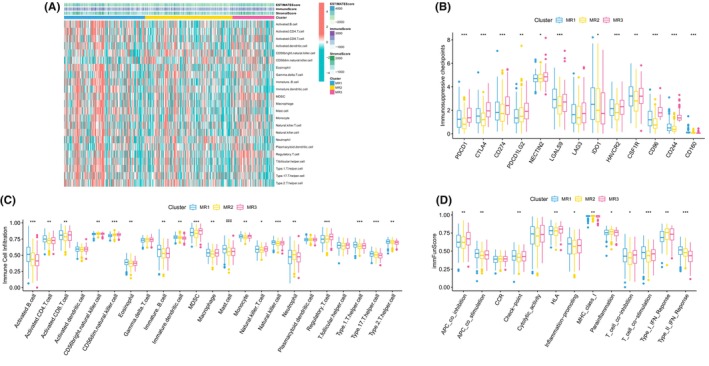
Immune infiltration characteristics of M2 macrophage‐related clusters. (A) Distribution of immune cells and TME scores among M2 macrophage‐related clusters; (B) differential expression of immunosuppressive checkpoints among the M2 macrophage‐related clusters; (C) expression differences of immune cells among the M2 macrophage‐related clusters; (D) expression differences of immune function scores among the M2 macrophage‐related clusters. * = *P* < 0.05; ** = *P* < 0.01; *** = *P* < 0.001.

Next, we examined the differences in immune infiltration among the M2 macrophage‐related clusters. The heatmap visually represented the distribution of immune cell expression and TME scores across the clusters (Figure 2A). Moreover, most immunosuppressive checkpoints were significantly overexpressed in MR3 (Figure 2B). We observed that immune cells exhibited the highest expression in MR1 and the lowest expression in MR3 (Figure 2C). Additionally, APC co‐stimulation, checkpoint, inflammation‐promoting and HLA pathways were enriched in the MR3 cluster (Figure 2D).

### 3.3 Construction and prognostic analysis of the M2 macrophage‐related signature

**FIGURE 3:**
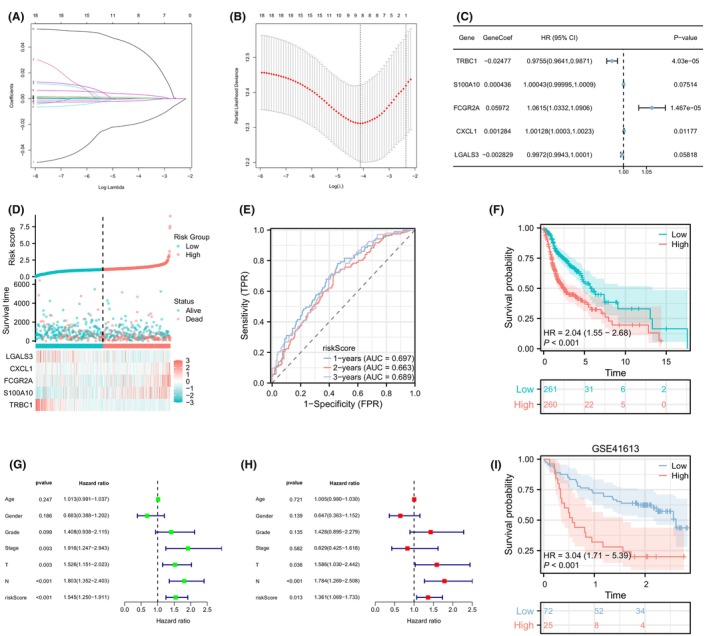
Establishment of the M2 macrophage‐related signature (MRS). (A) Least absolute shrinkage and selection operator (LASSO) regression analysis of the 18 prognostic M2 macrophage‐related genes (MRGs); (B) LASSO coefficients for the nine prognostic M2 MRGs; (C) multivariate Cox regression analysis for the five prognostic M2 MRGs; (D) distribution of gene expression and survival status in the M2 MRS. (E) ROC curves for the M2 MRS at 1, 2, and 3 years; (F) Kaplan–Meier (KM) survival analysis for the M2 MRS; (G, H) univariate and multivariate independent prognostic analyses for the M2 MRS; (I) KM survival analysis for the M2 MRS in the GSE41613 dataset.

To enhance robustness of our analysis, we employed LASSO and multivariate Cox regression analyses on 18 prognosis‐related MRGs, leading to the identification of five robust MRGs for constructing the MRS (Figure 3A–C). Analysis of the survival status distribution revealed that the high MRS group had a worse prognosis (Figure 3D). The MRS exhibited an area under the curve (AUC) predictive value of 0.697, 0.663, and 0.689 for the 1, 2, and 3‐year survival rates, respectively (Figure 3E). KM survival curve demonstrated that MRS was associated with a decreased chance of survival (Figure 3F). Univariate and multivariate independent prognostic analyses indicated that the MRS could function as an independent predictor (Figure 3G, H). Next, we validated the prognostic features of MRS in the GSE41613 datasets. The KM survival curve revealed that the high MRS group was associated with the poorer prognosis in the GSE41613 (Figure 3I).

### 3.4 Identification of immune infiltration characteristics of M2 macrophage‐related signature

**FIGURE 4:**
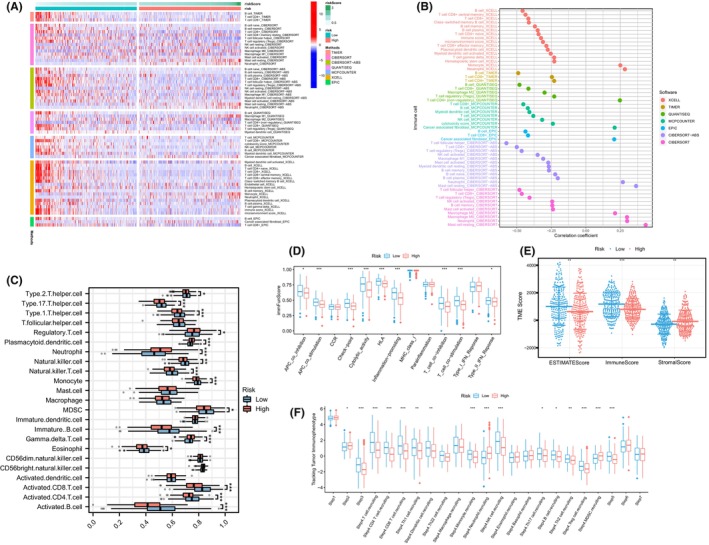
Immune infiltration characteristics of the M2 macrophage‐related signature (MRS). (A) Expression of immune cell in MRS by multiple algorithms; (B) correlation of immune cells and the MRS; (C) differential expression of immune cells within the MRS; (D) differential expression of immune function scores within the MRS; (E) differences in TME scores between different MRS groups; (F) differential expression of the MRS across different tumour immunophenotypes. * = *P* < 0.05; ** = *P* < 0.01; *** = *P* < 0.001.

The heatmap showcased the expression of immune cells in MRS groups across multiple algorithms (Figure 4A). Across various algorithms, the MRS was significantly negatively correlated with multiple immune cells (Figure 4B). Furthermore, compared to the high MRS group, most immune cells exhibited significant overexpression in the low MRS group (Figure 4C). Analysis of the immune function pathway revealed significantly higher expression of T cell co‐stimulation, APC co‐stimulation and Type II IFN Response in the low MRS group (Figure 4D). Moreover, all TME scores (ESTIMATE Score, Immune Score, and Stromal Score) were significantly overexpressed in the low MRS group (Figure 4E). To gain deeper insights into the involvement of immune cells in the progression of HNSC, we acquired immune activity scores at different stages from the TIP database. Figure 4F demonstrated that the low MRS group exhibited a significantly higher frequency of immune cells, suggesting a potential association between the MRS and immune cell activity in HNSC.

### 3.5 Correlation analysis of M2 macrophage‐related signature and clinicopathological stage

We conducted an extensive assessment of the correlation between the MRS and clinicopathological characteristics, including expression, distribution and survival. There were significant statistical differences in MRS expression across different clinicopathological stages, with MRS expression escalating as the clinicopathological stage advanced (Figure S1A). Additionally, the high MRS group exhibited a significantly higher proportion of advanced clinicopathological stages (Figure S1B). A thorough analysis of survival differences among MRS groups within distinct clinicopathological stages highlighted the poorer prognosis associated with the high MRS group (Figure S1C).

### 3.6 Tumour mutation burden characteristics and drug sensitivity of M2 macrophage‐related signature

**FIGURE 5:**
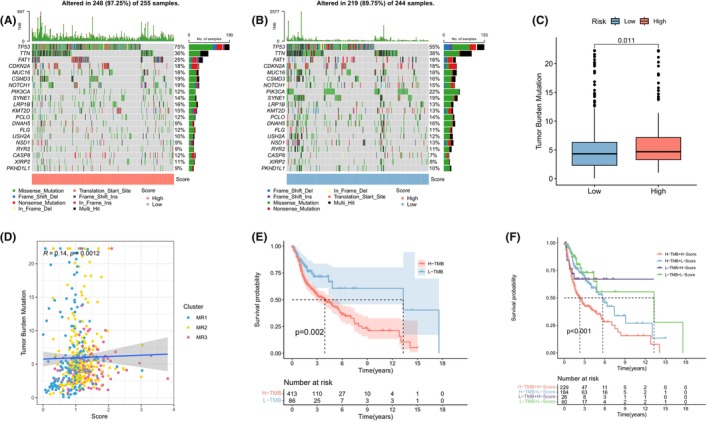
Tumour mutation characteristics of the M2 macrophage‐related signature (MRS). (A, B) Somatic mutations in high and low MRS groups. (C) Differential expression of TMB in M2 MRS groups. (D) Correlation analysis between TMB and the M2 MRS. (E) Kaplan–Meier curve analysis for TMB groups. (F) Survival analysis of combined TMB and the M2 MRS.

The mutation rate was 97.25% in the high MRS group and 89.75% in the low MRS group (Figure 5A, B). Tumour mutation burden (TMB) was notably higher in the high MRS group (Figure 5C). Furthermore, a positive correlation was observed between MRS and TMB (Figure 5D). KM survival curve indicated that high TMB was associated with a poorer prognosis (Figure 5E). An analysis combining TMB and MRS survival further confirmed that the high‐HRS + high‐TMB group had the worst prognosis, thereby validating the earlier results (Figure 5F). To assess association between the MRS and HNSC resistance, we analysed differences in sensitivity to nine drugs. The IC50 values for Rapamycin, Methotrexate, Gefitinib and Tipifarnib were higher in the high MRS group (Figure S2A–D). Conversely, the low MRS group exhibited higher IC50 values for Lapatinib, Cisplatin, Bleomycin, Imatinib and Erlotinib, suggesting that these five drugs may be more suitable for patients with a higher MRS (Figure S2E–I).

### 3.7 Correlation analysis of M2 macrophage‐related genes and clinicopathological stage

**FIGURE 6:**
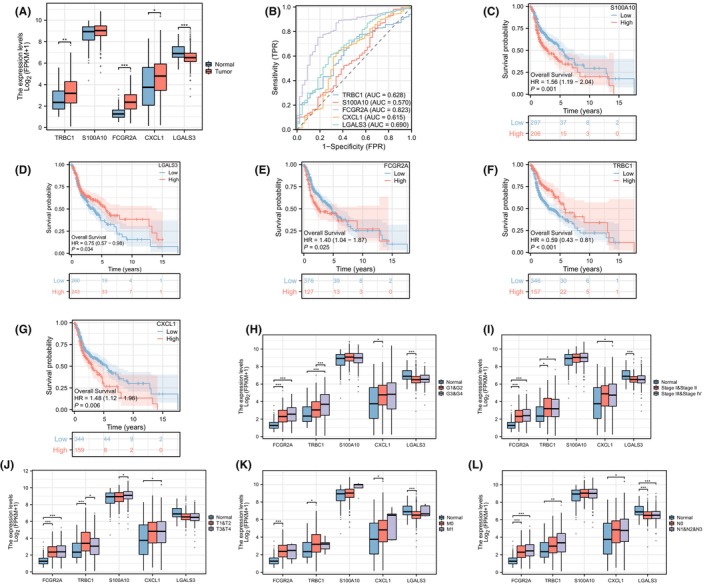
Prognostic and clinicopathological features of five modelled genes. (A) Expression differences of five modelled genes between cancer and precancerous tissues. (B) ROC curves for the five modelled genes. (C–G) Survival analyses for the five modelled genes. (H–L) Differential expression of the five modelled genes across different clinicopathological phases (H: Grade, I: Stage, J: T, K: M, L: N). * = *P* < 0.05; ** = *P* < 0.01; *** = *P* < 0.001.

In order to explore the potential mechanisms of MRGs in HNSC progression, we assessed the relationship between the five modelled MRGs and clinicopathological variables. Compared to normal tissues, TRBC1, FCGR2A and CXCL1 were significantly overexpressed in HNSC tissues, while LGALS3 exhibited the opposite pattern (Figure 6A). The AUC of the five modelled MRGs was presented in Figure 6B, with FCGR2A having the highest AUC value. The survival differences in overall survival (OS, overall survival is a measure of the length of time individuals or patients survive from a defined starting point (such as diagnosis or treatment initiation) until death from any cause) were analysed between the high and low groups. KM survival curves indicated that S100A10, FCGR2A and CXCL1 were associated with a poor prognosis, while TRBC1 and LGALS3 were positively related to patient prognosis (Figure 6C–G). Furthermore, the expressions of LGALS3 gradually decreased, while the expressions of FCGR2A, TRBC1, S100A10 and CXCL1 increased with the advancement of clinicopathological stage (Figure 6H–L).

### 3.8 Expression landscape of macrophage‐related genes in multiple cells

**FIGURE 7:**
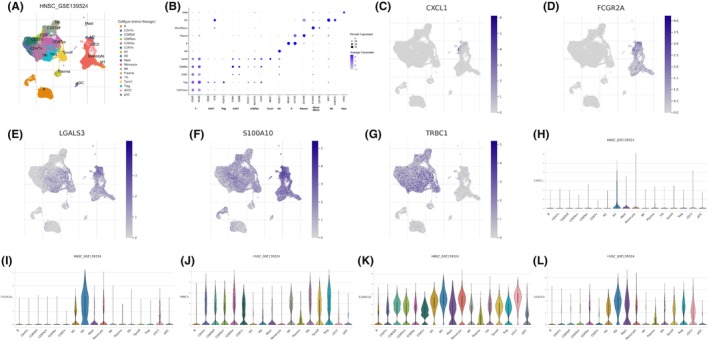
Single cell sequencing analysis of five modelled genes. (A) Composition and distribution of single cells from GSE139324; (B) the expression of typical marker genes in different cells; (C–L) the distribution and amount of five modelled genes (CXCL1, FCGR2A, LGALS3, S100A10 and TRBC1) expression in distinct cells. * = *P* < 0.05; ** = *P* < 0.01; *** = *P* < 0.001.

We examined the expression distribution of multiple cell subgroups in the GSE139324 dataset (Figure 7A). The cells were classified into different cell lines and labelled with the expression of typical marker genes (Figure 7B). CXCL1 was highly enriched in monocytes, FCGR2A in M2 macrophages, LGALS3 in macrophages, monocytes and regulatory T (Treg) cells, S100A10 in macrophages, monocytes and mast cells and TRBC1 in Treg, Tfh, CD8Tex and CD8Tn cells (Figure 7C–L).

### 3.9 Identification of immune infiltration characteristics of FCGR2A

**FIGURE 8:**
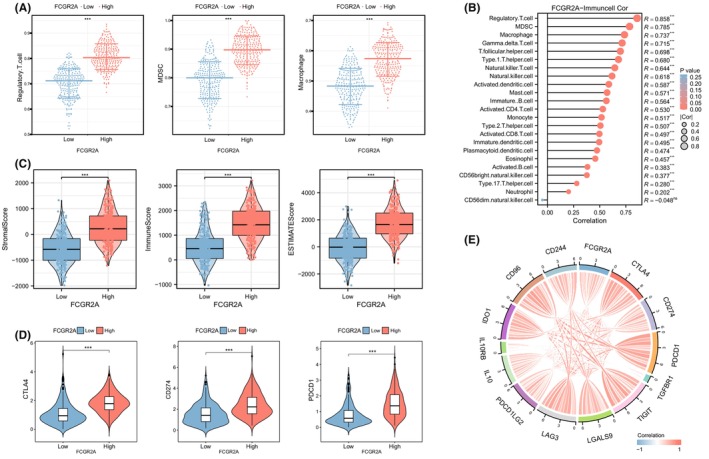
Immune infiltration characteristics of FCGR2A. (A) Differential expression of immunosuppressive cells in FCGR2A groups; (B) correlation analysis between immune cells and FCGR2A; (C) TME scores expression difference in FCGR2A groups; (D) expression differences of immunosuppressive checkpoints in FCGR2A groups; (E) correlation analysis between immunosuppressive checkpoints and FCGR2A.

Further investigations were conducted to explore the correlation between FCGR2A and immune cells, as well as immune checkpoints. The expression of immunosuppressive cells, such as macrophages, myeloid‐derived suppressor cells (MDSCs), and T cells, was higher in high FCGR2A group (Figure 8A). A significant positive correlation between FCGR2A and immunosuppressive cells was observed (Figure 8B). Immune microenvironmental characteristics of FCGR2A based on immune score, stromal score, and estimated score in HNSC tissues indicated a significant positive correlation with FCGR2A (Figure 8C). Additionally, CTLA4, PDCD1 and CTLA4 were all highly expressed in high FCGR2A group (Figure 8D). FCGR2A exhibited a significant positive correlation with various immune‐suppressive checkpoints (Figure 8E), suggesting a close association between FCGR2A and the establishment of an immunosuppressive microenvironment.

## 4 DISCUSSION

Studying and treating HNSC present a significant challenge due to its classification as a distinct group of cancers originating from various anatomical sites, associated with different risk factors, and exhibiting diverse molecular pathologies.^21^, ^22^ Technological advancements have greatly assisted in the treatment of HNSC patients through surgery and chemoradiotherapy.^23^ However, the high recurrence rate of HNSC has resulted in unsatisfactory prognostic survival rates.^21^, ^24^ In addition to mutations in tumour cells, the composition of the TME plays a pivotal role in treatment response and patient prognosis in HNSC.^25^, ^26^ M2 macrophages, which are involved in development of various tumours, have a significant influence on the immune microenvironment.^27^ Therefore, our study extended to analyse mechanisms of MRGs in HNSC and explore new biomarkers to guide clinical decision‐making and improve the prognosis of HNSC patients.

TAMs are significant components of the TME and have a complex role in tumour growth.^28^ In the TME, TAMs can transform into M2‐like TAMs under the influence of cytokines, thereby promoting tumour proliferation and metastasis.^29^, ^30^ Monocytes secrete chemokines, including CXCL12, during tumour progression, which further promotes the transition from M1 macrophages to M2 macrophages, ultimately supporting the tumour, inducing angiogenesis, and promoting immunosuppression.^31^, ^32^, ^33^ Studies have consistently demonstrated that high expression of M2 TAMs is significantly associated with poor prognosis in various tumours, such as pancreatic ductal adenocarcinoma (PDAC),^34^ glioblastoma^35^ and bladder cancer.^36^ The crucial role of M2 TAMs in tumour proliferation and progression underscores their potential as a therapeutic target, warranting ongoing research. However, the specific mechanisms and prognostic models of M2 TAMs in HNSC remain largely unexplored.

As is know that HNSC is an immunoresponsive tumour with high heterogeneity and metastasis potential, which leads to its unique immunological characteristics in terms of pathogenesis and treatment.^6^ Research revealed that in TME, invasive immune cells play a key role in tumour proliferation, metastasis and anti‐cancer immune regulation, and are extremely important therapeutic targets.^37^ We also know that the TME in HNSC contains transformed cells admixed with immune cells and stromal cellular elements. Extensive research on TME has revealed a crucial role of the tumour‐infiltrating immune cells in tumour dissemination, relapse, metastasis and therapeutic response to immunotherapy. Moreover, immunoinfiltration analysis is an important part of bioinformatics analysis.^38^, ^39^

In our study, we constructed three M2 macrophage‐related clusters based on the expression of 18 MRGs, each exhibiting distinct prognostic and immune properties. In recent years, immunotherapy has emerged as a prominent treatment approach for HNSC. Trials investigating immunotherapy targets in HNSC have yielded significant insights, and TAM‐related treatment may represent a promising strategy in the future. Given the critical role of the immune system in antitumor responses in HNSC, we conducted a comprehensive assessment of immune infiltrate characteristics using various algorithms. Analysing the differences in immune and biological pathways among the clusters revealed distinct infiltration characteristics of immune cells in TME. MR1 exhibited enrichment in immune cell infiltration and immune‐related pathways, suggesting an immunoinflammatory type. MR2 showed enrichment in innate immune cell infiltration and stroma‐related pathways, indicative of an immune exclusion type. MR3, on the other hand, displayed a lack of immune cell infiltration, signifying an immune desert type. However, despite the significant enrichment of innate immune cells in MR2, this cluster had a poor prognosis. It has been observed that immune‐rejected tumours exhibit immune cell infiltration confined to the matrix surrounding tumour cells, implying T cell inhibition within the tumour microenvironment.^40^ Additionally, the matrix activation pathway was significantly enriched in MR2, including ECM receptor interactions, cell cycle and tight junctions. Therefore, we hypothesized that the anti‐tumour effect of immune cells was suppressed by the activation of intermediates within the MR2 cluster. Subsequently, we constructed the MRS and validated its stability in predicting patient prognosis through univariate and multifactorial independent prognostic analyses. In the TME, infiltrating immune cells played a pivotal role in tumour proliferation, migration and the regulation of anti‐cancer immune responses, making them crucial therapeutic targets.^37^ TME scores and proportion of immunosuppressive cell infiltration were higher in the high MRS group, which corresponded to worse prognosis. In summary, the M2 macrophage‐related prognostic model served as a vital indicator for evaluating patient prognosis and immune response, guiding the formulation of personalized treatment strategies for HNSC patients.

FCGR2A belongs to the immunoglobulin receptor gene family.^41^, ^42^ FCGR2A binds to the Fc fragment of IgG2 antibodies and is highly expressed in macrophages, lymphocytes and other immune cells, regulating cell recognition, phagocytosis and cytotoxicity.^43^ Research has indicated that FCGR2A can promote antigen presentation, immune response expression, and tumour metastasis and invasion by inducing antibody‐dependent phagocytosis.^44^ Additionally, FCGR2A is significantly overexpressed in various cancers and associated with poor prognosis, such as clear cell renal cell carcinoma,^45^ malignant glioma^46^ and oesophageal squamous cell carcinoma.^47^ Our analysis revealed a significant overexpression of FCGR2A in HNSC, particularly in advanced clinicopathological stages. FCGR2A showed a positive correlation with immunosuppressive cells and immunosuppressive checkpoints. Thus, we speculated that FCGR2A may influence the immune microenvironment, promoting progression of HNSC and it could potentially function as an early diagnostic target for HNSC.

While our research provided valuable insights into importance of M2 macrophages in HNSC and addresses critical need for validated indicators in HNSC research, it does have certain limitations. First, we employed traditional LASSO and multivariate Cox analyses to construct M2 macrophage‐related prognostic model. Although these methods have been widely accepted and utilized in numerous studies, future research may benefit from more advanced techniques and methods. Additionally, due to the limitation of TCGA data, we lack other parameters, such as CT images, to further validate our model.

## 5 CONCLUSION

M2 macrophages have a profound impact on the development of HNSC, underscoring their critical role in the disease. The MRS demonstrated remarkable accuracy in evaluating patient prognosis and immune characteristics, offering valuable insights for clinical decision‐making. Additionally, FCGR2A has emerged as a potential therapeutic target in HNSC, as it appeared to be involved in establishment of an immunosuppressive microenvironment. These findings hold promise for the development of novel treatment strategies in HNSC.

## AUTHOR CONTRIBUTIONS

**Siyuan Wu:** Data curation (lead); formal analysis (lead); investigation (lead); methodology (lead); writing – original draft (equal). **Xiaozhi Lv:** Resources (lead); software (lead); supervision (lead); writing – original draft (equal). **Haigang Wei:** Validation (lead); visualization (lead); writing – original draft (equal). **Jialin Wu:** Data curation (supporting); formal analysis (supporting); funding acquisition (supporting); writing – review and editing (equal). **Shiwei Liu:** Methodology (supporting); project administration (supporting); resources (supporting); software (supporting); writing – review and editing (equal). **Xia Li:** Resources (supporting); software (supporting); supervision (supporting); writing – review and editing (equal). **Jing Song:** Supervision (supporting); validation (supporting); visualization (supporting); writing – review and editing (equal). **Chen Zou:** Conceptualization (supporting); supervision (supporting); visualization (supporting); writing – review and editing (supporting). **Yilong Ai:** Conceptualization (lead); funding acquisition (lead); methodology (lead).

## FUNDING INFORMATION

This work was supported by the Guangdong Basic and Applied Basic Research Foundation (grant number 2022A1515140098) and Scientific Research Project of Department of Education of Guangdong Province (Project Number: 2023ZDZX2057).

## CONFLICT OF INTEREST STATEMENT

The authors declare no conflict of interests.

## Supporting information


Figure S1.

Figure S2.

Table S1.

## Data Availability

The data that support the findings of this study are openly available in TCGA, GEO and TISCH2 datasets.
