# Supplementary figures and images for: Integrated analysis of single‐cell RNA‐seq and bulk RNA‐seq unravels the molecular feature of M2 macrophages of head and neck squamous cell carcinoma
Source: J Cell Mol Med. 2024 Feb 23;28(5):e18083. doi: 10.1111/jcmm.18083 (PMC10902578; doi:10.1111/jcmm.18083)

A

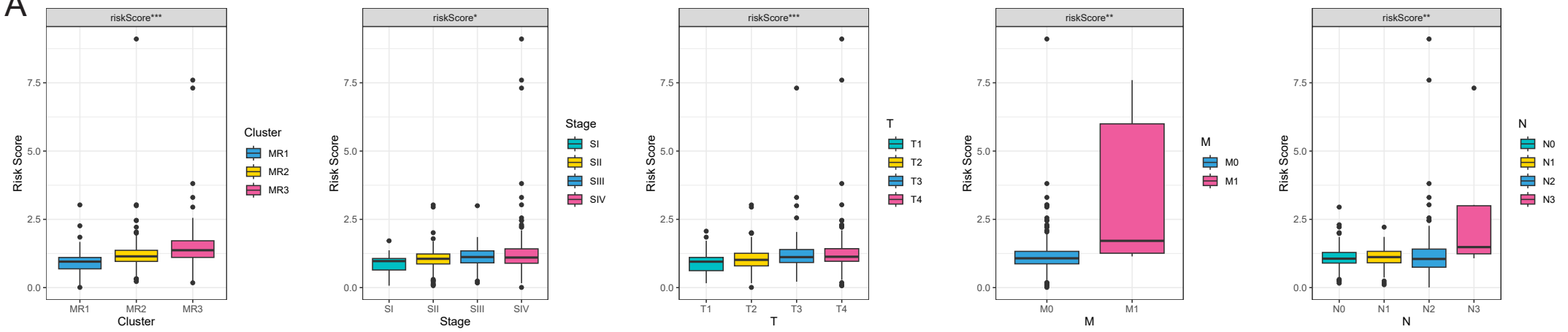

B

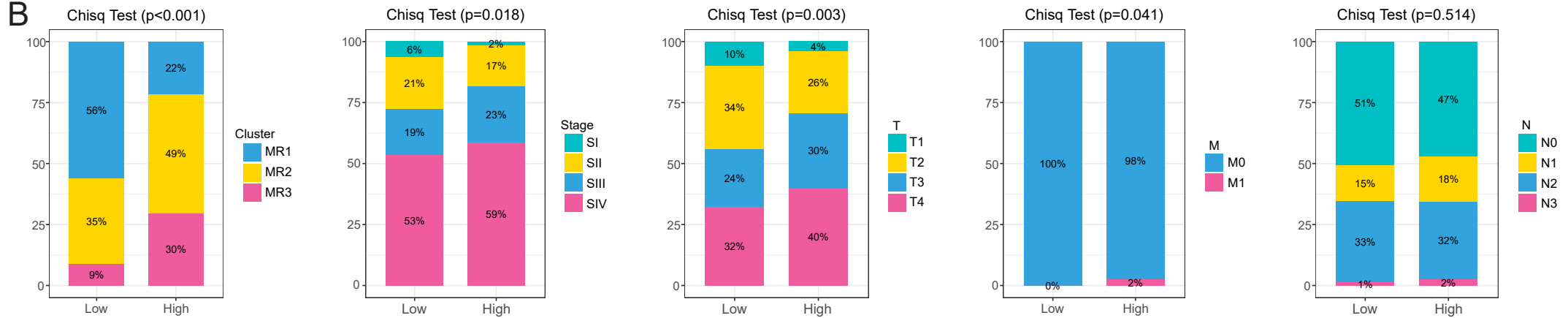

C

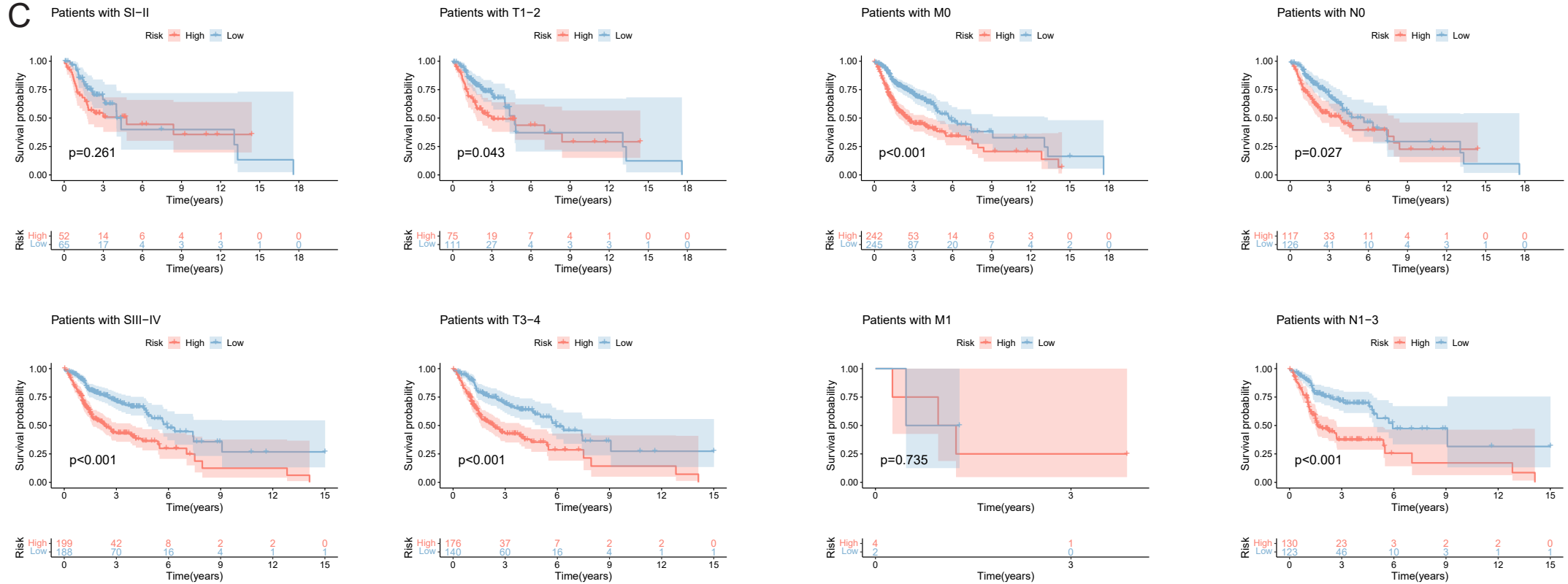

Supplement: Supplementary file 1 — Figure S1. [file JCMM-28-e18083-s002.pdf]

**A**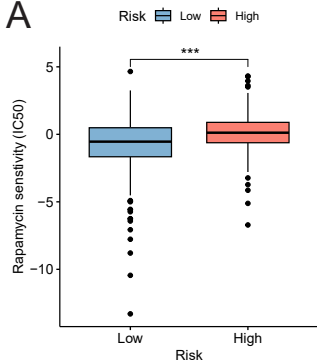**B**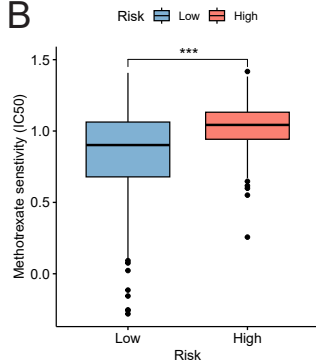**C**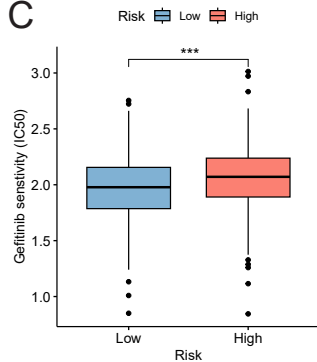**D**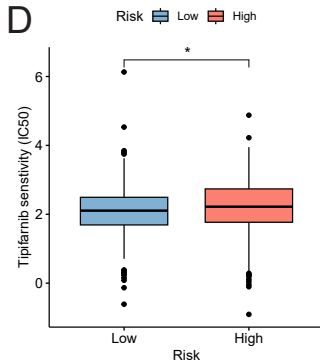**E**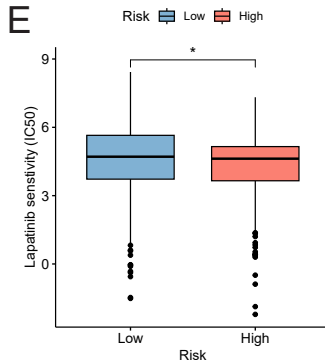**F**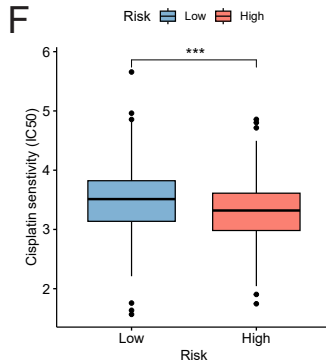**G**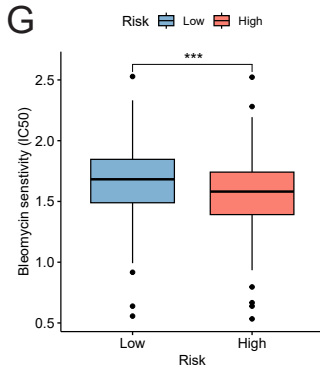**H**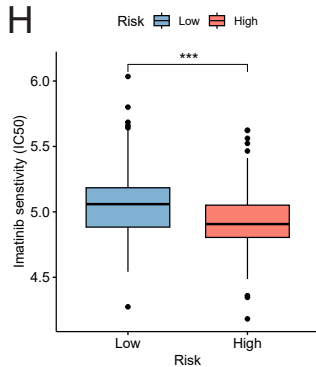**I**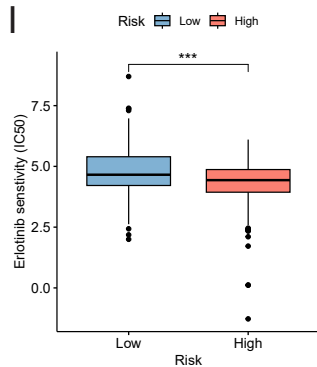

Supplement: Supplementary file 2 — Figure S2. [file JCMM-28-e18083-s003.pdf]
