# Supplementary material for: Integrated analysis of single‐cell RNA‐seq and bulk RNA‐seq unravels the molecular feature of M2 macrophages of head and neck squamous cell carcinoma
Source: J Cell Mol Med. 2024 Feb 23;28(5):e18083. doi: 10.1111/jcmm.18083 (PMC10902578; doi:10.1111/jcmm.18083)
Supplement: Supplementary file 3 — Table S1. [file JCMM-28-e18083-s001.docx]

| **List of abbreviations** |
| --- |
| Differentially expressed genes (DEGs) |
| Gene Ontology (GO) |
| Gene Set Variation Analysis (GSVA) |
| Half maximum inhibitory concentration (IC50) |
| Head and neck squamous cell cCarcinoma (HNSC) |
| Kaplan-Meier (KM) |
| Kyoto Encyclopedia of Genes and Genomes (KEGG) |
| Least absolute shrinkage and selection operator (LASSO) |
| M2 macrophage-related genes (MRGs) |
| M2 macrophage-related signature (MRS) |
| myeloid-derived suppressor cells (MDSCs) |
| Overall survival (OS) |
| Pancreatic ductal adenocarcinoma (PDAC) |
| Receiver operating characteristic (ROC) |
| Regulatory T (Treg) |
| The Cancer Genome Atlas (TCGA) |
| Tracking Tumor Immunophenotype (TIP) |
| Tumor Immune Single-cell Hub 2 (TISCH2) |
| Tumor microenvironment (TME) |
| Tumor mutation burden (TMB) |
